# Rhinophyma-Like Cutaneous Leishmaniasis due to *Leishmania aethiopica* Treated Successfully with Liposomal Amphotericin B

**DOI:** 10.4269/ajtmh.18-0578

**Published:** 2019-02

**Authors:** Michael Constantin Kirchberger, Stefan Schliep, Christian Bogdan

**Affiliations:** 1 Department of Dermatology, Universitätsklinikum Erlangen and Friedrich-Alexander-Universität (FAU) Erlangen-Nürnberg, Erlangen, Germany;; 2 Institute of Clinical Microbiology, Immunology and Hygiene, Universitätsklinikum Erlangen and Friedrich-Alexander-Universität (FAU) Erlangen-Nürnberg, Erlangen, Germany

**Figure 1.**
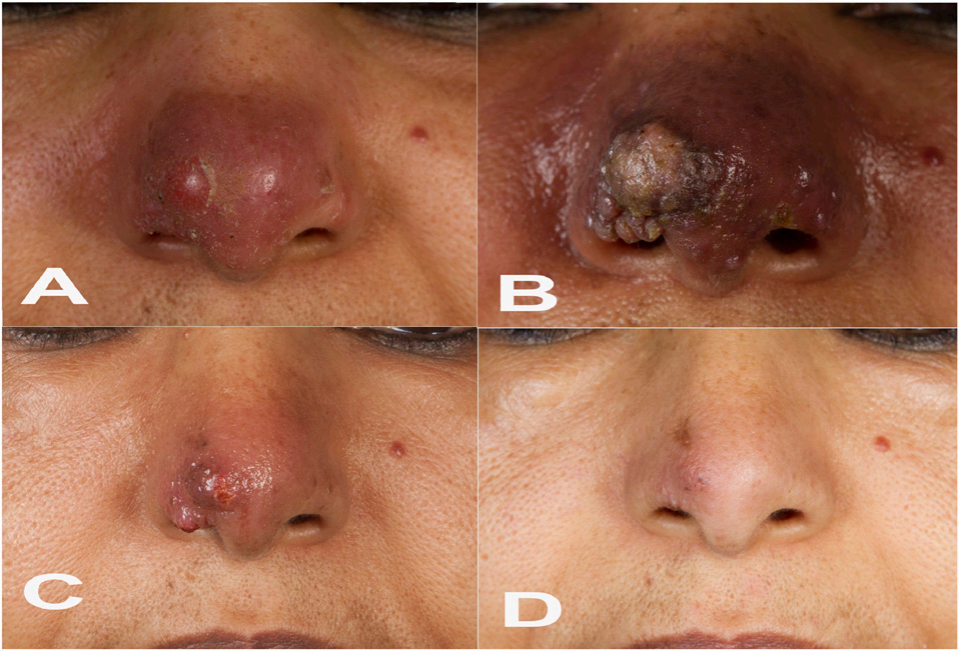
(**A**) Nodular and erythematous lesion of the nose before antiparasitic treatment. (**B**) Rhinophyma-like appearance of the nasal skin lesion 2 weeks after the first liposomal amphotericin B (LAmB) treatment cycle. (**C**) Regression of the nasal skin lesion 12 weeks after the second LAmB cycle. (**D**) Healed lesion 13 months after the second LAmB cycle. This figure appears in color at www.ajtmh.org.

A 57-year-old woman from Ethiopia, who has been a permanent resident in Germany for the past 30 years, presented with a nodular and erythematous skin lesion on her nose that had evolved 4 months ago (Figure 1A). She had previously undergone unsuccessful local and systemic antibiotic treatment (most likely with fusidic acid and oral cephalosporins, respectively). Cutaneous leishmaniasis (CL) was suspected based on the patient’s visit to Ethiopia 8 months earlier. A skin biopsy was cultured in modified Schneider’s insect cell medium,^1^ which yielded the growth of *Leishmania* promastigotes. The parasites were identified as *Leishmania* (*L.*) *aethiopica* by three different methods (mini-exon polymerase chain reaction [PCR] plus restriction fragment length polymorphism [RFLP] analysis^2^; Hsp70 PCR plus RFLP analysis^3^; and *Leishmania* cytochrome b sequencing^4^). The patient was hospitalized to rule out visceral and mucosal manifestations. Because of the complex location of the skin lesion which bears the risk of disfiguring scars and tissue destruction, the patient was treated with liposomal amphotericin B (LAmB) over a period of 10 days starting with 3 mg/kg body weight (210 mg/day). As she complained of vertigo and nausea, the dosage had to be reduced to 1.5 mg/kg body weight after 4 days. After 3 weeks, the patient unexpectedly developed multiple livid papules resembling rhinophyma, a condition otherwise seen in patients with rosacea and characterized by hypertrophy of the sebaceous glands of the nose (Figure 1B). A second cycle of LAmB with a reduced dosage (1.5 mg/kg body weight) was administered again for 10 days. During the following months, the severe nasal lesion gradually regressed (Figure 1C) and finally healed (Figure 1D), with a surprisingly positive cosmetic outcome that was highly satisfying for the patient. The initial clinical exacerbation might have resulted from the known release of proinflammatory cytokines in response to LAmB and/or from an overshooting T-cell response to *Leishmania* antigens following LAmb-mediated parasite destruction.

Therapeutic options with proven efficacy for CL caused by *L. aethiopica* are limited to pentavalent antimonials, pentamidine, and cryotherapy.^5^ However, toxicity or treatment failures are frequently observed and randomized controlled trials comparing different treatment strategies are lacking. Because of our positive experience with LAmB in complicated cases of CL elicited by *Leishmania major*^6^ or *Leishmania tropica* (C. Bogdan, unpublished), we decided to apply LAmB, which was also successfully used in an earlier case of *L. aethiopica* CL.^7^
